# Simulating recurrent event data with hazard functions defined on a total time scale

**DOI:** 10.1186/s12874-015-0005-2

**Published:** 2015-03-08

**Authors:** Antje Jahn-Eimermacher, Katharina Ingel, Ann-Kathrin Ozga, Stella Preussler, Harald Binder

**Affiliations:** Institute of Medical Biostatistics, Epidemiology and Informatics, University Medical Center Johannes Gutenberg University Mainz, Obere Zahlbacher Str. 69, Mainz, 55131 Germany

**Keywords:** Andersen-Gill, Recurrent events, Recurrent failure times, Simulation, Total time, Calendar time

## Abstract

**Background:**

In medical studies with recurrent event data a total time scale perspective is often needed to adequately reflect disease mechanisms. This means that the hazard process is defined on the time since some starting point, e.g. the beginning of some disease, in contrast to a gap time scale where the hazard process restarts after each event. While techniques such as the Andersen-Gill model have been developed for analyzing data from a total time perspective, techniques for the simulation of such data, e.g. for sample size planning, have not been investigated so far.

**Methods:**

We have derived a simulation algorithm covering the Andersen-Gill model that can be used for sample size planning in clinical trials as well as the investigation of modeling techniques. Specifically, we allow for fixed and/or random covariates and an arbitrary hazard function defined on a total time scale. Furthermore we take into account that individuals may be temporarily insusceptible to a recurrent incidence of the event. The methods are based on conditional distributions of the inter-event times conditional on the total time of the preceeding event or study start. Closed form solutions are provided for common distributions. The derived methods have been implemented in a readily accessible R script.

**Results:**

The proposed techniques are illustrated by planning the sample size for a clinical trial with complex recurrent event data. The required sample size is shown to be affected not only by censoring and intra-patient correlation, but also by the presence of risk-free intervals. This demonstrates the need for a simulation algorithm that particularly allows for complex study designs where no analytical sample size formulas might exist.

**Conclusions:**

The derived simulation algorithm is seen to be useful for the simulation of recurrent event data that follow an Andersen-Gill model. Next to the use of a total time scale, it allows for intra-patient correlation and risk-free intervals as are often observed in clinical trial data. Its application therefore allows the simulation of data that closely resemble real settings and thus can improve the use of simulation studies for designing and analysing studies.

**Electronic supplementary material:**

The online version of this article (doi:10.1186/s12874-015-0005-2) contains supplementary material, which is available to authorized users.

## Background

Recurrent event data are multivariate failure time data where the individuals experience repeated occurrences of the same type of event. In clinical applications, recurrent events are often particular medical conditions, such as hospitalizations due to a particular disease, cardiovascular events, epileptic seizures, episodes of multiple sclerosis or falls in elderly people. Many survival models have been proposed to handle recurrent event data [1], and simulations are commonly used to investigate statistical methods or to plan the sample size of a clinical trial [2]. A survey of PubMed indicates 36 articles within the last five years that feature the term *recurrent event* and *simulation* in the title or abstract.

Whereas some authors have demonstrated the need to investigate statistical methods under different event generation processes and correlation structures [3,4], little attention has been given to the time scale that is applied for subsequent events. In particular, simulation studies often generate data from a *gap time perspective*, where the time and thus the risk process is reset after each event, which simplifies the simulation process. Conversely, in many clinical applications a *total time perspective* is appropriate, i.e. where the hazard for experiencing a particular recurrent medical condition depends on the time since some starting point. To improve the accordance between the simulation models applied to investigate statistical techniques and the statistical models used to analyze clinical data, we have derived a flexible simulation algorithm that implements a total time perspective.

The definition of time that is used as the argument of the hazard function is a fundamental step in modeling data and affects the statistical results and their interpretation [5,6]. The starting point 0 in a total time perspective may be the onset of a disease, the beginning of some treatment, entering a clinical trial or birth (age-dependent risks) [7,8]. A total time scale is, for example, commonly applied when analyzing the efficacy of pneumococcal vaccines to prevent recurrent episodes of acute otitis media [9-12] because the risk of acute otitis media is known to be age-dependent [13,14]. Schneider *et al.* encourage the use of a total time scale when re-estimating the sample size for trials in relapsing multiple sclerosis [15], as total time trends of the event rates seem to be present [8]. Some authors use the term counting process time [6] or calendar time [5] to further specify the risk intervals that are to be used in the regression analysis. The time *scale* is the same as total time. A total time scale (counting process) also underlies the Andersen-Gill model, probably the most frequently applied model used to analyze recurrent failure time data in medical science. Conversely, in simulation studies often a gap time scale is used [3,4,6,16], sometimes by defining constant hazards. This may be due to a lack of information on how to simulate recurrent event data using a total time scale as opposed to published simulation algorithms for gap time [3,4] and other multivariate [17,18] or univariate [19] failure time data.

Furthermore, in simulation designs, the individuals are usually assumed to be continuously at risk for experiencing recurrent events. In real-life situations, individuals might be temporarily insusceptible to a recurrent incidence of the event (risk-free intervals). An example are relapsing diseases where effective treatments are available that prevent disease progression, but the treatments can only be discontinuously administered because of adverse side effects. Individuals experience disease relapses outside the treatment courses but are considered to be insusceptible to events under treatment.

The aim of this article is to provide a general approach for simulating recurrent event data when considering an Andersen-Gill model with a total time scale. We allow for arbitrary time-varying hazard functions, risk-free intervals, and incorporate inter-patient heterogeneity by including covariates and frailty terms.

The article is organized as follows: We start with clinical examples that characterize settings where total time models should be considered. Then, in the Methods section, we introduce the recurrent event model, derive the simulation algorithm and provide closed form solutions. In the Results section we demonstrate the implementation and illustrate the use of our methods on power and sample size planning for trials with complex recurrent event data. We discuss our methods and results and finally we finish the article with concluding remarks.

## Examples for total time scale settings

We illustrate the rationale behind the use of a total time scale on two clinical examples. In addition to a total time model, we will further assume that increments in the frequency of events over time do not depend on previous events (Andersen-Gill model). Only in the case of a constant hazard rate does this model reduce to a gap time model due to the memorylessness of the exponential distribution. When analyzing recurrent failure time data, individuals usually are assumed to be continuously at risk for experiencing events during follow-up. Different circumstances can prevent individuals from experiencing events within certain intervals within the observational period. These risk-free intervals have to be considered in the statistical model. In both examples risk-free intervals are present.

### Episodes of otitis media

**Figure 1:**
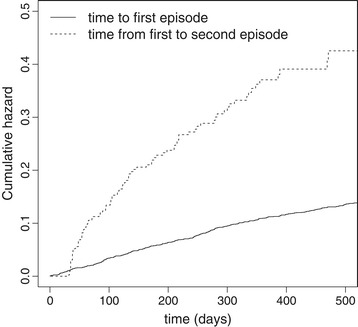
**Cumulative hazards in POET data.** Cumulative hazard for the time from 2 weeks after the third vaccine dose to first clinical episode of AOM and for the time from first to second clinical episode of AOM. Control group only of the randomized controlled clinical POET trial.

Acute otitis media (AOM) is one of the most commonly diagnosed childhood infections. The disease is most prevalent in children younger than two years of age. The efficacy of a conjugate pneumococcal vaccine to decrease the attack rate of AOM has been evaluated in a randomized controlled clinical trial on 4968 children. The trial has been designed and conducted by GlaxoSmithKline Biologicals and has been reported fully elsewhere [12]. It was done according to the Declaration of Helsinki and the protocol was reviewed and approved by the appropriate independent ethics committees or institutional review boards (NCT00119743). Infants aged between 6 weeks and 5 months were enrolled after informed consent had been obtained from a parent or legal guardian. For the 2452 children in the control group, who received a Hepatitis A vaccination, Figure 1 shows the cumulative hazard rates for the time from 2 weeks after the third vaccine dose (at 5 months of age) to the first clinical episode of AOM and for the gap time between the first and the second clinical episode of AOM. The cumulative hazards strongly differ from each other; this is contradictory to results we would expect in a gap time model. In addition to the known age-dependency of the risk for AOM [13,14], this calls for a total time modeling approach. It should be noted, that differences in gap time hazards can also result for other reasons, such as a violation of the independent increment assumption and/or confounding. Here, an episode of AOM is not assumed to affect the risk for subsequent events, at least in the long-run. Confounding might be present as children with a history of AOM may be, in general, more prone to experience episodes of AOM due to physiological or social circumstances. We will not cover confounding in the present manuscript. Interestingly, we observe a zero hazard for the gap time to the second event within the first 30 days. A new episode of otitis media was judged to have started only if more than 30 days had elapsed since the beginning of a previous episode. Thus, children are risk-free 30 days after each event by definition.

### Falls in elderly people

According to the World Health Organization (WHO) falls are the second leading cause of accidental or unintentional injury deaths worldwide. Adults older than 65 suffer the greatest number of fatal falls. Recently, the fall rate in elderly Australian adults was evaluated over a three year follow-up period [20]. The fall rate increased within three years from 0.33 falls per person-year to 0.55 falls per person-year. The difference in fall rates demonstrates the age-dependent risk to fall and thus the need of a total time modeling approach. In contrast, in a gap time model, the yearly fall rate would be expected to not differ between both observational periods. If a fall is followed by a period of hospitalization or bed rest, further falls will most probably not occur within these periods. As a consequence, patients are to be considered not at risk for further falls until they have restarted usual living.

## Methods

Consider the situation where individuals are followed for the times of occurrence of some recurrent event and a total time scale shall be used. We define *T*_*i*_ as the time from starting point 0 to occurrence of the *i*-th event. Let *N*(*t*)=*#*{*i*;*T*_*i*_≤*t*} denote the counting process representing the number of events experienced before time *t*. Assuming that prior events do not affect the risk for future events, the hazard process of *N*(*t*) is given by

(1) $$ \lambda(t) \mathrm{d}t = E\left[N(t+\mathrm{d}t)-N(t-)|F_{t^{-}}\right] = E\left[\mathrm{d}N(t)|F_{t^{-}}\right]  $$

with $\phantom {\dot {i}\!}F_{t^{-}}$ being the history up to time *t*. The cumulative hazard function is defined by $\Lambda (t)={\int _{0}^{t}} \lambda (s) \mathrm {d}s$.

### Distributional derivations

As recurrent events are naturally ordered, event times *T*_*i*_ can be derived from the inter-event times *U*_*i*_:=*T*_*i*_−*T*_*i*−1_ with *T*_0_=0 by $\phantom {\dot {i}\!}T_{i}=\sum _{k=1}^{i} U_{k}$. Therefore we aim to identify a method for simulating inter-event times. For that, we will first identify the distributions of inter-event times for an arbitrary hazard process of *N*(*t*). As the risk for events depends on total time, the distribution of an inter-event time depends on the time of the preceeding event unless we deal with the simple situation of constant hazards. Therefore, we consider the distributions of inter-event times conditional on the time of the preceeding event. Let *U*_*i*_|*T*_*i*−1_=*t* denote these conditional inter-event times. The conditional hazard function $\tilde {\lambda }^{i}$ of *U*_*i*_|*T*_*i*−1_=*t* can be derived for *i*>1 by

$${\fontsize{9.5}{6} \begin{aligned} \tilde{\lambda}^{i}(u|T_{i-1}=t) \mathrm{d}u &= P(u\leq U_{i} < u+\mathrm{d}u | U_{i} \geq u, T_{i-1}=t)\\ &= P(u+t\leq T_{i} < u+t+\mathrm{d}u | T_{i} \geq u\\ &\quad+t, T_{i-1}=t)\\ &= E[\mathrm{d}N(u+t)| T_{i} \geq u+t, T_{i-1}=t]\\ &= \lambda(u+t)\mathrm{d}u \end{aligned}} $$

and $\tilde {\lambda }^{1}(u)=\lambda (u)$.Accordingly we can derive the cumulative hazard of *U*_*i*_|*T*_*i*−1_=*t* for *i*>1 by

$$\begin{array}{@{}rcl@{}} \tilde{\Lambda}^{i}(u|T_{i-1}=t) &=& {\int_{0}^{u}} \tilde{\lambda}^{i}(s|T_{i-1}=t)\mathrm{d}s \\ &=& {\int_{0}^{u}} \lambda(s+t)\mathrm{d}s \\ &=& \Lambda(u+t)-\Lambda(t) \end{array} $$

and $\tilde {\lambda }^{1}(u)=\lambda (u)$.

Note that the conditional (cumulative) hazards $\tilde {\lambda }^{i}$ and $\tilde {\lambda }^{i}$ do not depend on *i*. This is caused by the assumption that the risk to experience events is not affected by previous events. Thus, we can define

(2) $$\begin{array}{@{}rcl@{}} \tilde{\Lambda}_{t}(u)&:=&\tilde{\Lambda}^{i}(u|T_{i-1}=t) =\Lambda(u+t)-\Lambda(t)  \end{array} $$

For a specific time to recurrent event model, closed form solutions can be found for $\tilde {\Lambda }_{t}$ derived from *λ*. Examples will be presented in the section ‘Closed form solutions for common distributions’.

Transferring the arguments of Bender *et al*. [19] to conditional survival distributions we reveal for *i*>1 that the conditional random variable $(\exp (-\tilde {\Lambda }_{t}(U_{i}))|T_{i-1}=t)$ follows a uniform distribution on the interval from 0 to 1. Therefore

$$\begin{array}{@{}rcl@{}} U_{i}|T_{i-1}=t &\sim & \tilde{\Lambda}_{t}^{-1}(-\log(A)) \end{array} $$

with *A*∼*U*[0,1]. For *i*=1, *U*_1_=*T*_1_∼*Λ*^−1^(− log(*A*)).

### The recursive simulation algorithm

Using the derivations in the ‘Distributional derivations’ section, a random realization (*t*_*i*_) of (*T*_*i*_) can be generated as follows

- Specify the hazard *λ*(*t*) as a function of total time.
- Derive $\tilde {\Lambda }_{t}$ and $\tilde {\Lambda }_{t}^{-1}$ according to (2).
- Simulate independent random numbers *a*_*i*_ following a uniform distribution on the interval from 0 to 1.
- Apply the recursive algorithm
  - *i*=1: *t*_1_=*Λ*^−1^(− log(*a*_1_))
  - $i\rightarrow i+1: \;\;t_{i+1} = t_{i} + \tilde {\Lambda }_{t_{i}}^{-1}(-\log (a_{i+1}))$.

As illustrated in the section ‘Examples for total time scale settings’, an event *i* might be followed by a period of length *d* within which no further events are expected. Thus, the hazard within [ *T*_*i*_,*T*_*i*_+*d*] is assumed to be zero. The recursive simulation algorithm can easily be adopted to consider risk-free intervals by applying

- $i\rightarrow i+1: \;\; t_{i+1} = t_{i}+d + \tilde {\Lambda }_{t_{i}+d}^{-1}(-\log (a_{i+1}))$

in step 4 of the algorithm. Here, in analogy to (2), $\tilde {\Lambda }_{t_{i}+d}^{-1}(u)$ is defined as *Λ*(*u*+*t*_*i*_+*d*)−*Λ*(*t*_*i*_+*d*).

### Closed form solutions for common distributions

Step 2 of the recursive simulation algorithm requires knowledge of the (inverse) conditional cumulative hazard function of the inter-event times, $\tilde {\Lambda }_{t}(u)$ and $\tilde {\Lambda }_{t}^{-1}(u)$, which can be derived from the hazard process *λ*(*t*). We will exemplarily derive these functions for the Weibull, Log normal and Gompertz distribution [21]. For the Weibull hazard with *λ*(*t*)=*λ*·*ν*·*t*^*ν*−1^ and *Λ*(*t*)=*λ*·*t*^*ν*^ we derive

$$\begin{array}{@{}rcl@{}} \tilde{\Lambda}_{t}(u)&=&\lambda\cdot ((t+u)^{\nu} - t^{\nu})\\ \tilde{\Lambda}^{-1}_{t}(u)& = & \left(\frac{u+\lambda \cdot t^{\nu}}{\lambda}\right)^{1/\nu} -t \end{array} $$

For the Log normal hazard with $\lambda (t) = \frac {1}{t\sigma } \phi \left (\frac {\log (t)-\mu }{\sigma }\right)/ \Phi \left (-\frac {\log (t)-\mu }{\sigma }\right)$ and $\Lambda (t)=-\log \left (1-\Phi \left (\frac {\log (t)-\mu }{\sigma }\right)\right)$ with *ϕ*(·) and *Φ*(·) being the probability density function and the cumulative distribution function of the standard normal distribution, respectively, we derive

$${ \fontsize{9}{6}\begin{aligned} \tilde{\Lambda}_{t}(u)& = \log\left(\frac{1-\Phi\left(\frac{\log(t)-\mu}{\sigma}\right)}{1-\Phi\left(\frac{\log(t+u)-\mu}{\sigma}\right)}\right)\\ \tilde{\Lambda}^{-1}_{t}(u)& =\exp \left(\! -\Phi^{-1}\left(\! \exp \left(\!\log \left(\! 1-\Phi \left(\! \frac{\log(t)-\mu}{\sigma }\! \right)\! \right) -u \!\right) \!\right)\! \cdot \right.\\&\left.{\vphantom{\frac{\log(t)-\mu}{\sigma }}}\quad\sigma + \mu \right) -t \end{aligned}} $$

And for the Gompertz hazard with *λ*(*t*)=*λ* exp(*αt*) and $\Lambda (t)=\frac {\lambda }{\alpha }(\exp (\alpha t)-1)$ we derive

$$\begin{array}{@{}rcl@{}} \tilde{\Lambda}_{t}(u)& = & \frac{\lambda}{\alpha}\left(\exp(\alpha(u+t))-\exp(\alpha t)\right)\\ \tilde{\Lambda}^{-1}_{t}(u)& = & \frac{1}{\alpha} \log\left(\frac{\alpha}{\lambda} u +\exp(\alpha t) \right)-t \end{array} $$

### Simulation in the Andersen-Gill model

A common total time model allowing for fixed covariates is the Andersen-Gill model [1,22], where the intensity process is modeled as

(3) $$\begin{array}{@{}rcl@{}} Y(t) \cdot \lambda(t) = Y(t) \cdot \lambda_{0}(t) \cdot \exp(\beta^{t} X), \end{array} $$

with *X* and *β* being *p*-dimensional vectors of fixed covariates and regression coefficients, respectively. *Y*(*t*) is the predictable process that equals one as long as the individual is under observation and at risk for an event. The baseline hazard *λ*_0_(*t*) can be an arbitrary non-negative function, e.g. defined by the Weibull or Gompertz parametrization as specified in the section ‘Closed form solutions for common distributions’, which leads to the hazard functions

(4) $$\begin{array}{@{}rcl@{}} \lambda(t) = \lambda\cdot \nu \cdot t^{\nu-1}\cdot\exp(\beta^{t} X) \end{array} $$

and

(5) $$\begin{array}{@{}rcl@{}} \lambda(t) = \lambda \cdot \exp(\alpha t)\cdot\exp(\beta^{t} X), \end{array} $$

respectively. In combination with the Andersen-Gill model, we do not use the Log normal parametrization due to its non-proportional behavior of the hazards.

The Andersen-Gill model assumes within-patient independency in the sense that the hazard for experiencing an event does not depend on previous events and that all individuals have a common baseline hazard *λ*_0_(*t*). Yet for many situations it may be more realistic that the baseline risk varies between individuals, for example if there are unobserved or unobservable characteristics affecting the time to event outcome. This can be modeled by introducing a frailty variable *Z* to the hazard function with *E*(*Z*)=1 and *Var*(*Z*)=*θ*, defining the frailty model [23]:

(6) $$\begin{array}{@{}rcl@{}} \lambda(t) = \lambda_{0}(t) \cdot Z \cdot \exp(\beta^{t} X) \end{array} $$

When fitting a misspecified Andersen-Gill model (3) to data that follow the frailty model model (6) (and thus are subject to unobserved inter-patient heterogeneity) regression coefficients are estimated unbiasedly [16]. However, to account for the intra-patient correlation that is caused by the frailty term, standard errors used for calculating statistical tests and confidence intervals have to be replaced by robust versions [1,24] to control level *α*.

The derived recursive simulation algorithm can easily be extended to simulate recurrent event data following an Andersen-Gill model with time-invariant covariates (3) or the frailty model extension (6). Although the hazard function will differ for each realization (*x*,*z*) of (*X*,*Z*), we can simplify the derivations of $\tilde {\Lambda }_{t}(u)$ and $\tilde {\Lambda }^{-1}_{t}(u)$ for different (*x*,*z*) and thus step 2 of the simulation algorithm. Let $\Lambda _{0}(t)={\int _{0}^{t}} \lambda _{0}(s)ds$ denote the cumulative baseline hazard. Assuming we have derived the (inverse) conditional cumulative baseline hazard of inter-event times $\tilde {\Lambda }_{0,t}(u)$ and $\tilde {\Lambda }_{0,t}^{-1}(u)$ corresponding to *λ*_0_(*t*) (*X*=0,*Z*=1) once, the (inverse) conditional cumulative hazard of inter-event times corresponding to *λ*(*t*) (6) can be derived for any realization (*x*,*z*) of (*X*,*Z*) by

(7) $$\begin{array}{@{}rcl@{}} \tilde{\Lambda}_{t}(u)&=& \Lambda(u+t)-\Lambda(t)  \\ &=& z\cdot \exp(\beta^{t} x)\cdot(\Lambda_{0}(u+t)-\Lambda_{0}(t))  \\ &=& z\cdot \exp(\beta^{t} x) \cdot\tilde{\Lambda}_{0,t}(u)  \\ \tilde{\Lambda}_{t}^{-1}(u) &=& \tilde{\Lambda}_{0,t}^{-1}(z^{-1}\cdot \exp(-\beta^{t} x)\cdot u) \end{array} $$

Simulation of *T*_*i*_ for different realizations of (*x*,*z*) can therefore be performed by first deriving $\tilde {\Lambda }_{0,t}(u)$ and $\tilde {\Lambda }_{0,t}^{-1}(u)$, then for each (*x*,*z*) calculating $\tilde {\Lambda }_{t}^{-1}(u)$ by (7) and afterwards applying step 3 and step 4 of the recursive simulation algorithm.

This approach will in particular simplify the simulation of data representing a sample of individuals each experiencing recurrent events and each differing in their covariate values. Examples will be presented in the section ‘Results’.

## Results

First, we will illustrate the implementation of the derived algorithm on two exemplary simulation studies. Thereafter, we will provide an example, that demonstrates the use of our methods for sample size planning.

### Implementation

As already noted by Bender *et al*. [19] random numbers following a *U*[0,1] distribution are frequently available in statistical program packages. Therefore, the recursive algorithm can easily be implemented within common software. We implemented the simulation algorithm in the open-source statistical environment R, version 3.1.0 (2014-04-10) [25] and provide the stand-alone R function as Additional file 1 with a detailed description as Additional file 2. The output dataset is provided in counting process style as required by most software packages when analysing data applying the Andersen-Gill model. We illustrate this implementation on two simulation studies. In the first study, we evaluate the effect of discontinuous risk intervals on the precision of cumulative hazard estimation. We randomly generate 1000 datasets, each with *N*=100 observational units experiencing events over a period of two years, that follow a Weibull distribution with scale $\lambda =4/\sqrt {2}$ and shape *ν*=0.5 by multiple calls of



A further 1000 datasets are generated with the same underlying distributional assumptions but incorporating the presence of risk-free intervals that follow events and last three months by multiple calls of



**Figure 2:**
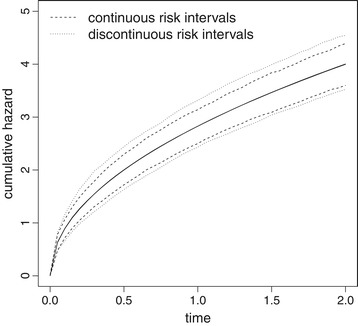
**Effect of risk-free intervals on cumulative hazard estimation.** Pointwise mean cumulative hazard estimates (solid lines) and corresponding 2.5*%* and 97.5*%* quantiles derived from 1000 simulated datasets with 100 observations each, that are observed over the follow-up period [0,2]. Data are generated without risk-free intervals (continuous risk intervals) and with risk-free intervals of length *d*=3/12 following each event (discontinuous risk intervals), respectively. Data distribution is Weibull with $\text {scale}=4/\sqrt {2}$ and shape=0.5. Mean estimates derived from the two simulation models are overlapping.

Figure 2 shows the pointwise mean cumulative hazard estimates with 2.5*%* and 97.5*%* quantiles as derived from the simulated data. As expected, risk-free intervals will not affect the point estimates if the statistical analysis method accounts for the discontinuous risk intervals. However, risk-free periods reduce the observed number of events which is the main determinant for efficiency in time to event analysis. As a consequence the precision of estimation is decreased as indicated by the broader interquantile range.

In a second simulation study, we evaluate the impact of unadjusted inter-patient heterogeneity on the bias and precision of regression parameter estimation when applying the Andersen-Gill model (3) for data analysis. Inter-patient heterogeneity is realized by including a random frailty term when simulating data. Independent datasets are randomly generated, each with *N*=100 observational units that experience events over time [0,2] following an event generation process according to model (6). We underlie a Weibull baseline hazard with scale $\lambda =4/\sqrt {2}$ and shape *ν*=0.5, incorporate a random Bernoulli distributed covariate X ∼*B*(1,0.5) with regression coefficient *β*=1 and a Gamma-distributed frailty term with mean 1 and variance *θ* [21]. For each *θ*∈{0,0.1,0.2,0.3,0.4,0.5} we randomly generate 1000 datasets by multiple calls of



**Figure 3:**
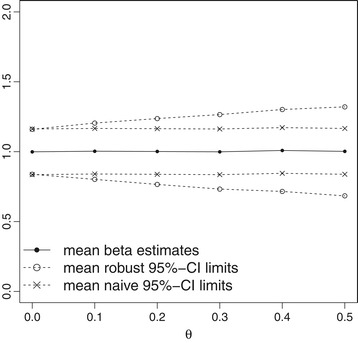
**Regression parameter estimation of Andersen-Gill model in the presence of unadjusted inter-patient heterogeneity.** Mean regression coefficient estimates with mean 95*%* confidence interval limits as derived from Andersen-Gill analysis applying naive and robust standard errors, respectively. Results are derived from 1000 simulated datasets each reflecting recurrent event data from 100 individuals with binary covariate *X* and regression coefficient *β*=1 and with gamma-distributed frailty term with mean 1 and variance *θ*, that reflects inter-patient heterogeneity. Baseline hazard is defined as Weibull with $\text {scale}=4/\sqrt {2}$ and shape=0.5.

The standard error of $\hat {\beta }$ is estimated using the second derivatives of the partial log likelihood (naive) and using robust sandwich estimates that take potential inter-patient heterogeneity into account (robust), respectively. Figure 3 demonstrates that regression parameters are estimated in an unbiased manner irrespective of the degree of inter-patient heterogeneity (*θ*). Naive standard errors do not account for *θ* and are therefore known to provide erroneously narrow confidence intervals and inflated type I errors. In contrast, the robust standard errors indicate that increased inter-patient heterogeneity is associated with an increased variability of effect estimates and therefore provide adjusted broader confidence intervals.

### Application: Sample size determination for complex recurrent event data

In the planning phase of a clinical trial the sample size *N*_0_ has to be determined, that is required to obtain a specific power under a prespecified clinically relevant effect size. Sample size formulas exist and can safely be applied for many trial designs and statistical tests. However, with increasing complexity of trial data, appropriate sample size formulas are often missing. For these situations simulation algorithms provide a useful statistical tool to derive the required sample size *N*_0_. Random samples each of size *N* are simulated and statistically analyzed while *N* varies iteratively until the sample power approximately coincides with the targeted power.

We will illustrate the use of the recursive simulation algorithm for sample size determination in a clinical trial with complex time to recurrent event data. As a hypothetical illustrating example, we consider a balanced randomized controlled trial aimed at investigating whether a particular intervention will reduce the incidence of falls in elderly people (section ‘Falls in elderly people’). Individuals are to be followed for a period of two years after starting the intervention. As the fall rate is expected to change with total time, the Andersen-Gill model with a single binary covariate indicating the randomized intervention will be applied, and the null hypothesis of no intervention effect, *H*_0_:{*β*=0}, is to be tested at a two-sided significance level of 5*%*. It is assumed that the two-year-incidence can be decreased from 3.72 in the control group to 2.74 in the intervention group. These assumptions are based on results observed in a controlled trial on vitamin D supplementation [26], where person-years rates of 1.86 and 1.37 have been observed in the control and vitamin D group, respectively. Furthermore, we assume that the risk to fall is expected to increase with time (see section ‘Falls in elderly people’), and therefore we assume the failure time data to follow a Weibull-distribution with shape parameter *ν*>1. We illustrate sample size derivations for an assumption of *ν*=2. The scale parameter is derived as 3.72/2^*ν*^=0.93 in the control group and 2.74/2^*ν*^=0.69 in the intervention group to be consistent with the two-year-incidence rates of 3.72 and 2.74. The hazard ratio between intervention and control group is 0.69/0.93=0.74. Furthermore, it has to be considered that some of the individuals will drop-out early for example due to death or the onset or deterioration of a disease that prevents further participation in the trial. For illustrative reasons, we assume a high lost to follow-up rate of 50*%* and assume this early drop-out to follow a uniform distribution on the interval [ 0,2]. If data follow this distributional pattern, known sample size formulas, for example as proposed by Bernardo and Harrington [27], can be applied indicating that a sample size of 160 individuals is required for the detection of a statistically significant intervention effect with a power of 80*%*.

However, closed and simple to apply sample size formulas often fail if the complexity of the data distribution increases. In the present clinical trial example, it must be further considered that falls can be followed by a period of hospitalization or bed rest. As a consequence, these patients are considered insusceptible to subsequent falls until they have restarted usual living (risk-free intervals). For illustrative reasons we consider two scenarios. In the first one we assume that falls are followed by relatively short risk-free intervals of length *d*=2 weeks with probability *p*=0.2. In the second scenario prolonged risk-free intervals of *d*=8 weeks are expected, which arise with a higher probability of *p*=0.5 after each event. According to the results of the section ‘Implementation’ (Figure 2), the presence of risk-free intervals affects the precision of parameter estimation and, as a consequence, the power of a trial. We do not know of a sample size formula that takes risk-free intervals into account and therefore apply the simulation algorithm to determine the required sample size. For each setting, 10000 datasets are randomly generated and statistically analyzed using the Wald test statistic within the Andersen-Gill approach. Simulation results indicate that a few small risk-free intervals only marginally affect the required sample size, whereas the presence of longer and more frequent risk-free intervals increases the sample size requirements to N=184 individuals.

We further expect that data distribution and thus sample size requirements will be affected by unobserved inter-patient heterogeneity: The risk of falling is known to not only depend on known and measured covariates but also on further unknown or unobserved patient characteristics as for example neuromuscular functioning, bone fragility or the frequency of going outdoors. For this reason, we expect the fall rate to vary between individuals even if important covariates are taken into account. It is a known result that unobserved inter-patient heterogeneity causes intra-patient correlations and thus has to be considered in statistical analysis to control the type I error. For that, the use of robust standard errors has been recommended [1,24], but it is expected to increase sample size requirements according to the results of the section ‘Implementation’ (Figure 3). Therefore, we further adapt our simulation study and incorporate inter-patient heterogeneity using a Gamma-distributed random frailty term with mean 1 and variance *θ*. We run simulations for each *θ*∈{0,0.1,0.2,0.3,0.4,0.5} to reflect different degrees of heterogeneity. Results are summarized in Table 1 and demonstrate a relevant increase in the required sample size with increasing inter-patient heterogeneity.

**Table 1:** **Sample size calculation results**

	**Number of patients** ***N***_0_	
***θ***	***d*** **=2,** ***p*** **=0** ***.*** **2**	***d*** **=8,** ***p*** **=0** ***.*** **5**
0	160	184
0.1	204	226
0.2	252	274
0.3	296	320
0.4	340	366
0.5	380	422

The simulation algorithm was applied to derive the number of individuals required for 80% power applying the robust two-sided Wald test at a 5% significance level for different frailty variances ***θ*** and risk-free intervals of length *d* (weeks) following an event with probability *p*. Each result was derived from 10000 simulation runs.

Example code for derivation of these results is given in the Appendix along with the stand-alone R function simrec.

## Discussion

We have presented a general method for simulating recurrent event data when the hazard is defined on a total time scale. In particular, this method allows the simulation of recurrent event data following an Andersen-Gill model by incorporating fixed and random covariates. Our application demonstrates the use of the simulation design for planning a clinical trial, in particular if trial data are expected to be complex. We have also applied the simulation algorithm to evaluate the impact of risk-free intervals and unobserved inter-patient heterogeneity on time to event estimates. The proposed simulation algorithm can also be applied to simulate the recurrence process in joint models for recurrent events and a terminal event [28,29]. We have considered models with a total time scale as this will be reasonable in many clinical settings. Gap time models are defined by the distribution of the inter-event times, thus simplifying their simulation. Pietzner and Wienke [30] proposed a trend-renewal process to include both a total time scale and a gap time scale and Duchateau *et al.* [5] applied both time scales to analyze recurrent asthma events in a parametric frailty model. It will be interesting for further research to extend our simulation algorithm to incorporate more than one time scale. It should be noted that there is a long history of methods for simulating inhomogeneous Poisson processes, with time-scale-transformations of homogeneous Poisson processes [31] and thinning [32] being two prominent methods. Conversely, we have derived an algorithm to simulate the inter-event times. We think that this approach has two advantages that are relevant for medical science: first, risk-free intervals can be incorporated in the simulation design, which will be useful for many applications where individuals are temporarily insusceptible to a recurrent incidence of the event; and second, simulating inter-event times allows for simulating hazards that change with the number of previous events. Metcalfe [3] demonstrated the need to randomly generate these kind of data for the evaluation of statistical methods. We provided an R function simrec for simulating recurrent event data under a total time scale in the counting process style. This function fills the gap between existing R packages for simulating gap time models (covered by complex.surv.dat.sim [33]) and for simulating inhomogeneous Poisson processes using time scale transformations (covered by NHPoisson [34]). We aim to extend this function to an R package that will be available from the standard CRAN repository for the R environment.

## Conclusions

We have derived an algorithm for simulating data following an Andersen-Gill model defined on a total time scale. The use of a total time scale provides a better fit to many medical trial data than the commonly applied gap time simulation models. Furthermore, our method allows for a complex data structure by incorporating intra-patient correlation and/or risk-free intervals. Its application therefore allows the simulation of data that closely resemble real settings and thus can improve the use of simulation studies for designing and analysing studies.

## Additional files

Additional file 1
**Provides a readily accessible** R **function, that implements the derived technique for simulating recurrent event data following an Andersen-Gill model.** Number of individuals, minimum and maximum length of follow-up and censoring probability can be passed to the function as arguments. Furthermore, distributions for fixed and random covariates and the corresponding regression coefficients can be determined. The baseline hazard is to be specified either as Weibull, as Gompertz or, if no covariates are present, as Log normal and will be defined on a total time scale. Events might be followed by a risk-free interval of length *d* with a probability *p*, and these parameters can also be passed to the function as arguments. For example, for the simulation of 100 individuals with a follow-up of 2 time-units, a binomially distributed covariate, a random covariate and Weibull distributed event data, the function simrec.R can be called as:
simdata <- simrec(N=100,fu.min=2,fu.max=2, dist.x=~binomial~,par.x=0.5,beta=1, dist.z=~gamma~,par.z= *𝜃* , dist.rec=~weibull~,par.rec=c( $4/\sqrt {2},0.5$ ))
This call will provide a dataframe simdata that contains the simulated data in counting process style, where the covariate is denoted as x.V1. Then the data can be analysed by use of the coxph-function in the survival-package, where cluster(id) indicates that robust standard errors are computed for the model:
coxph(Surv(start, stop, status) ∼ x.V1 + cluster(id), data = simdata)
Further details are given in the manuscript and the annotated script with vignette.

Additional file 2
**Provides a detailed description of the provided** **R** function **simrec.R** **including some exemplarily** R **code for calling that function.**
