# Supplementary material for: Simulating recurrent event data with hazard functions defined on a total time scale
Source: BMC Med Res Methodol. 2015 Mar 8;15:16. doi: 10.1186/s12874-015-0005-2 (PMC4387664; doi:10.1186/s12874-015-0005-2)
Supplement: Additional file 2 — Provides a detailed description of the provided R function simrec.R including some exemplarily R code for calling that function. [file 12874_2015_5_MOESM2_ESM.pdf]

simrec: An R-Package for Simulation of Recurrent Event Data

Katharina Ingel
IMBEI Mainz

Stella Preussler
IMBEI Mainz

Antje Jahn-Eimermacher
IMBEI Mainz

Abstract

simrec allows simulation of recurrent event data following the multiplicative intensity model described in Andersen and Gill [1] with the baseline hazard being a function of the total/calendar time. To induce between-subject-heterogeneity a random effect covariate (frailty term) can be incorporated.

Keywords: recurrent event data, simulation, total-time model.

Description

This function allows simulation of recurrent event data following the multiplicative intensity model described in Andersen and Gill [1] with the baseline hazard being a function of the total/calendar time. To induce between-subject-heterogeneity a random effect covariate (frailty term) can be incorporated. Data for individual i are generated according to the intensity process

$$Y_i(t) \cdot \lambda_0(t) \cdot Z_i \cdot \exp(\beta^t X_i),$$

where X_i defines the covariate vector and β the regression coefficient vector. $\lambda_0(t)$ denotes the baseline hazard, being a function of the total/calendar time t, and $Y_i(t)$ the predictable process that equals one as long as individual i is under observation and at risk for experiencing events. Z_i denotes the frailty variable with $(Z_i)_i$ iid with $E(Z_i) = 1$ and $Var(Z_i) = \theta$. The parameter θ describes the degree of between-subject-heterogeneity. Data output is in the counting process format.

```
simrec(N, fu.min, fu.max, cens.prob = 0, dist.x = "binomial", par.x = 0, beta =
0, dist.z = "gamma", par.z = 0, dist.rec, par.rec, pfree = 0, dfree = 0)
```

Parameters

- `N` Number of individuals
- `fu.min` Minimum length of follow-up.
- `fu.max` Maximum length of follow-up. Individuals length of follow-up is generated from a uniform distribution on `[fu.min, fu.max]`. If `fu.min=fu.max`, then all individuals have a common follow-up.
- `cens.prob` Gives the probability of being censored due to loss to follow-up before `fu.max`. For a random set of individuals defined by a B(N,`cens.prob`)-distribution,

the time to censoring is generated from a uniform distribution on `[0, fu.max]`. Default is `cens.prob=0`, i.e. no censoring due to loss to follow-up.

- `dist.x` Distribution of the covariate(s) X. If there is more than one covariate, `dist.x` must be a vector of distributions with one entry for each covariate. Possible values are `"binomial"` and `"normal"`, default is `dist.x="binomial"`.
- `par.x` Parameters of the covariate distribution(s). For `"binomial", par.x` is the probability for $x = 1$. For `"normal"`, `par.x=c(`μ, σ`)` where μ is the mean and σ is the standard deviation of a normal distribution. If one of the covariates is defined to be normally distributed, `par.x` must be a list, e.g. `dist.x <- c("binomial", "normal")` and `par.x <- list(0.5, c(1,2))`. Default is `par.x=0`, i.e. $x = 0$ for all individuals.
- `beta` Regression coefficient(s) for the covariate(s) x. If there is more than one covariate, `beta` must be a vector of coefficients with one entry for each covariate. `simrec` generates as many covariates as there are entries in `beta`. Default is `beta=0`, corresponding to no effect of the covariate x.
- `dist.z` Distribution of the frailty variable Z with $E(Z) = 1$ and $Var(Z) = \theta$. Possible values are `"gamma"` for a Gamma distributed frailty and `"lognormal"` for a lognormal distributed frailty. Default is `dist.z="gamma"`.
- `par.z` Parameter θ for the frailty distribution: this parameter gives the variance of the frailty variable Z. Default is `par.z=0`, which causes $Z \equiv 1$, i.e. no frailty effect.
- `dist.rec` Form of the baseline hazard function. Possible values are `"weibull"` or `"gompertz"` or `"lognormal"`.
- `par.rec` Parameters for the distribution of the event data.
  If `dist.rec="weibull"` the hazard function is

  $$\lambda_0(t) = \lambda \cdot \nu \cdot t^{\nu-1},$$

  where $\lambda > 0$ is the scale and $\nu > 0$ is the shape parameter. Then `par.rec=c(`λ, ν`)`. A special case of this is the exponential distribution for $\nu = 1$.
  If `dist.rec="gompertz"`, the hazard function is

  $$\lambda_0(t) = \lambda \cdot \exp(\alpha t),$$

  where $\lambda > 0$ is the scale and $\alpha \in (-\infty, +\infty)$ is the shape parameter. Then `par.rec=c(`λ, α`)`.
  If `dist.rec="lognormal"`, the hazard function is

  $$\lambda_0(t) = \frac{1}{\sigma t} \cdot \frac{\phi(\frac{ln(t)-\mu}{\sigma})}{\Phi(\frac{-ln(t)-\mu}{\sigma})},$$

  where ϕ is the probability density function and Φ is the cumulative distribution function of the standard normal distribution, $\mu \in (-\infty, +\infty)$ is a location parameter and $\sigma > 0$ is a shape parameter. Then `par.rec=c(`μ, σ`)`. Please note, that specifying `dist.rec="lognormal"` together with some covariates does not specify the usual lognormal model (with covariates specified as effects on the parameters of the lognormal distribution resulting in non-proportional hazards), but only defines the baseline hazard and incorporates covariate effects using the proportional hazard assumtion.

- `pfree` Probability that after experiencing an event the individual is not at risk for experiencing further events for a length of `dfree` time units. Default is `pfree=0`.
- `dfree` Length of the risk-free interval. Must be in the same time unit as `fu.max`. Default is `dfree=0`, i.e. the individual is continously at risk for experiencing events until end of follow-up.

Output

The output is a `data.frame` consisting of the columns:

- `id` An integer number for identification of each individual
- `x` or `x.V1, x.V2, ...` - depending on the covariate matrix. Contains the randomly generated value of the covariate(s) X for each individual.
- `z` Contains the randomly generated value of the frailty variable Z for each individual.
- `start` The start of interval `[start, stop]`, when the individual starts to be at risk for a next event.
- `stop` The time of an event or censoring, i.e. the end of interval `[start, stop]`.
- `status` An indicator of whether an event occured at time `stop` (`status=1`) or the individual is censored at time `stop` (`status=0`).
- `fu` Length of follow-up period `[0,fu]` for each individual.

For each individual there are as many lines as it experiences events, plus one line if being censored. The data format corresponds to the counting process format.

Details

Data are simulated by extending the methods proposed by Bender et al [2] to the multiplicative intensity model.

Example

```
R> library(simrec)
R> ### Example:
R> ### A sample of 10 individuals
R>
R> N <- 10
R>
R> ### with a binomially distributed covariate with a regression coefficient
R> ### of beta=0.3, and a standard normally distributed covariate with a
R> ### regression coefficient of beta=0.2,
R>
R> dist.x <- c("binomial", "normal")
R> par.x  <- list(0.5, c(0,1))
```

```
R> beta   <- c(0.3, 0.2)
R>
R> ### a gamma distributed frailty variable with variance 0.25
R>
R> dist.z <- "gamma"
R> par.z  <- 0.25
R>
R> ### and a Weibull-shaped baseline hazard with shape parameter lambda=1
R> ### and scale parameter nu=2.
R>
R> dist.rec <- "weibull"
R> par.rec  <- c(1,2)
R>
R> ### Subjects are to be followed for two years with 20\% of the subjects
R> ### being censored according to a uniformly distributed censoring time
R> ### within [0,2] (in years).
R>
R> fu.min    <- 2
R> fu.max    <- 2
R> cens.prob <- 0.2
R>
R> ### After each event a subject is not at risk for experiencing further events
R> ### for a period of 30 days with a probability of 50\%.
R>
R> dfree <- 30/365
R> pfree <- 0.5
R>
R> simdata <- simrec(N, fu.min, fu.max, cens.prob, dist.x, par.x, beta,
+                      dist.z, par.z, dist.rec, par.rec, pfree, dfree)
R> print(simdata[1:10,])

##    id x.V1        x.V2         z     start      stop status fu
## 1   1    0  1.46292768 1.2572749 0.0000000 0.6404475      1  2
## 2   1    0  1.46292768 1.2572749 0.6404475 0.9298805      1  2
## 3   1    0  1.46292768 1.2572749 1.0120723 1.6661735      1  2
## 4   1    0  1.46292768 1.2572749 1.6661735 1.9501491      1  2
## 6   2    0 -0.01444291 0.7320683 0.0000000 0.5289349      1  2
## 7   2    0 -0.01444291 0.7320683 0.6111267 0.8005113      1  2
## 8   2    0 -0.01444291 0.7320683 0.8827031 1.3592523      1  2
## 9   2    0 -0.01444291 0.7320683 1.4414441 1.8065396      1  2
## 10  2    0 -0.01444291 0.7320683 1.8065396 2.0000000      0  2
## 11  3    0  1.90306577 0.8842771 0.0000000 1.4478808      1  2
```

References

1. Andersen P, Gill R (1982): Cox's regression model for counting processes: a large sample study. The Annals of Statistics 10:1100-1120

2. Bender R, Augustin T, Blettner M (2005): Generating survival times to simulate Cox proportional hazards models. Statistics in Medicine 24:1713-1723

Affiliation:

Katharina Ingel
Institute of Medical Biostatistics, Epidemiology and Informatics (IMBEI)
University Medical Center of the Johannes Gutenberg-University Mainz
55101 Mainz, Germany
E-mail: ingel@uni-mainz.de
URL: http://www.unimedizin-mainz.de/imbei
